# Nitroxyl Hybrids with Curcumin and Stilbene Scaffolds Display Potent Antioxidant Activity, Remodel the Amyloid Beta Oligomer, and Reverse Amyloid Beta-Induced Cytotoxicity

**DOI:** 10.3390/antiox13111411

**Published:** 2024-11-18

**Authors:** Madhu S. Budamagunta, Hidetoshi Mori, Joshua Silk, Ryan R. Slez, Balázs Bognár, Ulises Ruiz Mendiola, Tamás Kálai, Izumi Maezawa, John C. Voss

**Affiliations:** 1 Department of Biochemistry & Molecular Medicine, University of California, Davis, CA 95616, USA; msbudamagunta@ucdavis.edu (M.S.B.); jwsilk@ucdavis.edu (J.S.); rrslez@ucdavis.edu (R.R.S.); 2 Center for Genomic Pathology, University of California Davis, Sacramento, CA 95817, USA; 3 Paramag Biosciences Inc., 720 Olive Drive, Davis, CA 95616, USA; 4 Institute of Organic and Medicinal Chemistry, Faculty of Pharmacy, University of Pécs, Honvéd St. 1., H-7624 Pécs, Hungary; balazs.bognar@aok.pte.hu (B.B.); tamas.kalai@aok.pte.hu (T.K.); 5 János Szentágothai Research Center, Ifjúság St. 20., H-7624 Pécs, Hungary; 6 M.I.N.D. Institute and Department of Pathology and Laboratory Medicine, University of California Davis, Sacramento, CA 95817, USA; ruizmen@ucdavis.edu

**Keywords:** Alzheimer’s disease, oxidative stress, amyloid beta peptide, Aβ oligomer, protein misfolding, electron paramagnetic resonance spectroscopy, EPR, bifunctional drug, antioxidant, nitroxide

## Abstract

The disorder and heterogeneity of low-molecular-weight amyloid-beta oligomers (AβOs) underlie their participation in multiple modes of cellular dysfunction associated with the etiology of Alzheimer’s disease (AD). The lack of specified conformational states in these species complicates efforts to select or design small molecules to targeting discrete pathogenic states. Furthermore, targeting AβOs alone may be therapeutically insufficient, as AD progresses as a multifactorial, self-amplifying cascade. To address these challenges, we have screened the activity of seven new candidates that serve as Paramagnetic Amyloid Ligand (PAL) candidates. PALs are bifunctional small molecules that both remodel the AβO structure and localize a potent antioxidant that mimics the activity of SOD within live cells. The candidates are built from either a stilbene or curcumin scaffold with nitroxyl moiety to serve as catalytic antioxidants. Measurements of PAL AβO binding and remolding along with assessments of bioactivity allow for the extraction of useful SAR information from screening data. One candidate (HO-4450; PMT-307), with a six-membered nitroxyl ring attached to a stilbene ring, displays the highest potency in protecting against cell-derived Aβ. A preliminary low-dose evaluation in AD model mice provides evidence of modest treatment effects by HO-4450. The results for the curcumin PALs demonstrate that the retention of the native curcumin phenolic groups is advantageous to the design of the hybrid PAL candidates. Finally, the PAL remodeling of AβO secondary structures shows a reasonable correlation between a candidate’s bioactivity and its ability to reduce the fraction of antiparallel β-strand.

## 1. Introduction

The two neuropathological hallmarks of Alzheimer’s disease are beta amyloid (Aβ) plaques and neurofibrillary tangles (NFTs). Aβ is the product of the cleavage of the amyloid precursor protein (APP). The amyloid beta (Aβ) peptide plays a central role in the etiology of Alzheimer’s disease (AD), representing the earliest and most validated marker for the disease [1,2]. Targeting aggregates of Aβ currently serves as the only FDA-approved disease-modifying therapy for AD [3]. However, complications associated with immunotherapy against extracellular Aβ deposits present serious concerns surrounding their high risk of causing neurovascular events in patients [4]. The mitigation of adverse effects from immunotherapy might be achieved by combination therapy with small molecules that modulate contributing factors (e.g., neuroinflammation) or facilitate the clearance of complexes comprising immunoglobin and Aβ [5].

Small molecules have also been explored for their direct effect on Aβ oligomers (AβOs), which are soluble aggregates of the peptide associated with neurotoxicity [1,6]. Small molecules have advantages in terms of accessing both intracellular and extracellular AβO pools. Intraneuronal Aβ may play a key role in AD initiation, preceding both intracellular NFTs and extracellular amyloid deposits [7,8,9]. Previous data have suggested that the two pools (intracellular and extracellular) of Aβ seem to be in a dynamic equilibrium, and so targeting intracellular Aβ may also influence the levels of extracellular AβOs [8,10,11].

The multifactorial nature of AD is commonly recognized, requiring the involvement of several drug targets to modify disease initiation and progression. Oxidative stress, one of the earliest markers of AD progression [12,13], drives dysfunction along multiple fronts, including inflammation and the impairment of biomolecule function. For example, reactive oxygen species (ROS) arising from age-related mitochondrial dysfunction and endoplasmic reticulum stress propagate other pathways of AD pathogenesis, including microglial activation and Tau phosphorylation and misfolding [14]. Thus, oxidative stress is intimately connected to the cascade of degeneration, with ROS increasing Aβ production [15,16,17]. This relationship results in a vicious cycle between ROS and the accumulation of neurotoxic forms of AβO, both intracellularly and extracellularly.

**Figure 1:**
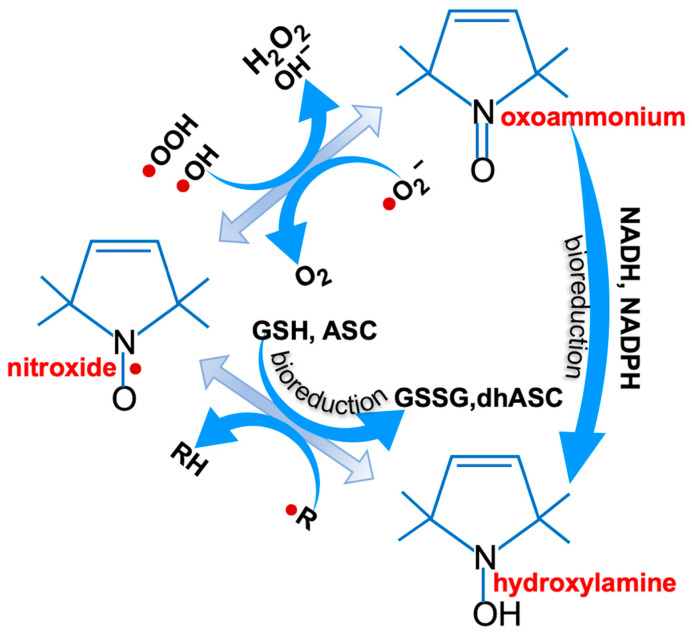
The potent radical scavenging of the nitroxide in cells. The N-oxyl cycles through alternative redox states. In the presence of the principal intracellular antioxidants glutathione (GSH) and ascorbate (ASC), the nitroxide undergoes bioreduction, enabling multiple rounds of PAL antioxidant activity.

To intervene in the self-amplifying cascade of AD progression, we have developed a class of bifunctional small molecules, Paramagnetic Amyloid Ligands (PALs), which have the potential to selectively bind and detoxify intracellular AβOs in addition to exerting potent and catalytic antioxidant activity [18,19,20,21,22,23]. Although oxidative stress is detrimental to all biomolecules, a particular concern is the resulting sidechain modifications that can potentiate Aβ toxicity [24,25,26,27]. Thus, in addition to altering peptide structure and assembly, the affinity of PALs for Aβ provides a mechanism with which to localize antioxidant functionality. This is capable of blocking oxidative modification [28]. PALs contain an aromatic component that binds to AβOs, inducing conformational adaptation and a nitroxyl moiety that imparts both paramagnetic and catalytic antioxidative properties. The antioxidant capacity of the added nitroxide moiety can mimic the antioxidant defense of superoxide dismutase (SOD) [29,30]. The nitroxide species can cycle within a redox cascade via the N-oxyl (nitroxyl), which is not oxidative to other biomolecules (lipids, proteins, DNA) (Figure 1). In the cytoplasm of cells, the N-O state of nitroxides N-O) is reduced to the N-OH state, which can be re-oxidized to N-O by ROS. In turn, hydroxyl and peroxyl radicals can oxidize the N-O state into oxoammonium (N=O), which is then able to remove superoxide and regenerate the N-O state. Subsequent reduction (e.g., by GSH) to N-OH allows for a single nitroxide to perform several rounds of scavenging.

Here, we report on the ability of a novel stilbene mimic PAL (HO-4897; PMT-304) to modulate AβOs’ structure, toxicity, and oxidative stress. In addition, we investigate the utility of previously synthesized paramagnetic analogs of curcumin [31] and resveratrol [32] regarding their potential to serve as bioactive PAL agents, capable of countering AD pathology. For each PAL candidate, a multidimensional measurement of AβO engagement, structural alteration, and cytotoxicity is reported, along with their relative antioxidant capacity. Finally, a preliminary pilot study of a selected stilbene-based PAL in the 5xFAD mouse model suggests the therapeutic activity of this candidate.

## 2. Materials and Methods

### 2.1. Materials

The amyloid beta (Aβ) peptide (1–40) was purchased from EZBiolab (Carmel, IN, USA). The Hoechst Blue 3342 nuclear stain and oligomer A11 polyclonal antibody were purchased from Thermo Fisher, USA. Opti-Minimal Essential Medium (OPTIMEM) was purchased from Invitrogen/Life Technologies, Carlsbad, CA, USA. PBS with pH 7.4 (-Calcium Chloride, -Magnesium Chloride), Opti-MEM^®^ I Reduced Serum Medium (no phenol red), DMEM (Dulbecco’s modified Eagle’s medium +4.5 g/L glucose, L-glutamine, and 110 mg/L sodium pyruvate), and Fetal Bovine Serum (FBS) were purchased from Gibco (Carlsbad, CA, USA). Trypan Blue Solution (0.4%) was purchased from Sigma-Aldrich.

Stilbene PALs HO-4408 (PMT-305), HO-4415 (PMT-306), and HO-4450 (PMT-307) were synthesized as previously described [32]. Curcumin PALs HO-4503 (PMT-701), HO-4507 (PMT-702), and HO-4757 (PMT-703) were synthesized as previously described [31]. Unless indicated, all other chemical reagents and solvents were purchased from Sigma-Aldrich (St. Louis, MO, USA).

### 2.2. Synthesis of HO-4897 (PMT-304)

**Scheme 1:**
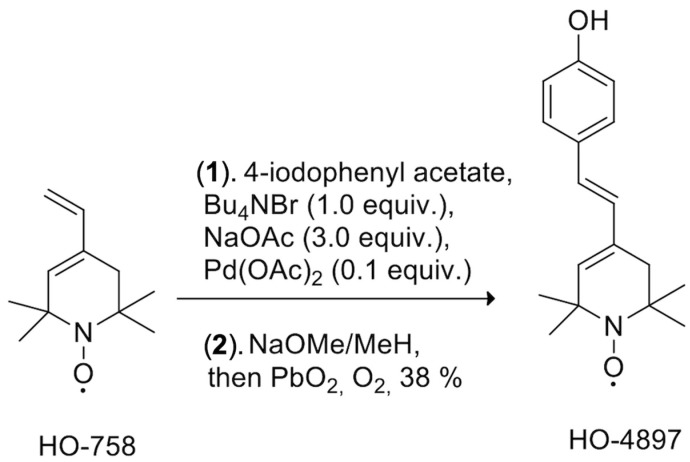
Synthesis of HO-4897.

The overall synthesis of (E)-4-(4-hydroxystyryl)-2,2,6,6-tetramethyl-1,2,3,6-tetrahydropyrine-1-yloxyl radical (HO-4897) is shown in Scheme 1.

To begin, we added Pd(OAc)_2_ (44 mg, 0.2 mmol) to a stirred, deoxygenated solution of HO-758 (360 mg, 2.0 mmol), 4-iodophenyl acetate (524 mg, 2.0 mmol), anhydr. NaOAc (492 mg, 6.0 mmol), and Bu_4_NBr (644 mg, 2.0 mmol) in anhydr. DMF (8 mL), and the mixture was heated in a sealed tube for 24 h at 90 °C. After cooling, the solvent was evaporated off, the residue was dissolved in MeOH (20 mL), freshly made NaOMe (2 mL MeOH +23 mg Na) was added, and the mixture was set aside for 2 h at 25 °C. After the solvents were evaporated off, the residue was partitioned between CHCl_3_ (25 mL) and sat. aq. NH_4_Cl (10 mL). The organic phase was separated, washed with 10% aq. Na_2_S_2_O_3_, and then dried with MgSO_4_. We added PbO_2_ (50 mg) to this solution and O_2_ was bubbled through the solution for 10 min. The mixture was filtered and evaporated, and the residue was purified by flash column chromatography (hexane/EtOAc) to furnish the title compound as an orange solid of 206 mg (38%), mp 67–68 °C, and R_f_: 0.34 (hexane/EtOAc, 2:1). IR (cm^−1^): 3266, 2975, 2906,1606, 1538, 1512. ^1^H NMR (CDCl_3_ + (PhNH)_2_) δ: 1.32 (s, 6H, 1 × C(C*H*_3_)), 1.38 (s, 6H, 1 × C(C*H*_3_)_2_), 2.40 (s, 2H, C*H*_2_), 5.64 (s, 1H, ring C*H*), 6.43 (d, 1*H*, *J* = 16 Hz, ArC*H*=), 6.68 (d, 1H, C*H*, *J* = 16 Hz, C*H*=CHAr), 6.82 (d, 2H, Ar*H*, *J* = 8 Hz), 7.34 (d, 2H, Ar*H*, *J* = 8 Hz). ^13^C NMR (CDCl_3_ + (PhNH)_2_) δ: 25.2 (s, 12, C(*C*H_3_)_2_), 26.0 (s, 2C, C(*C*H_3_)_2_), 39.3 (s, 1C, *C*H_2_), 57.6 (s, 1C, *C*(CH_3_)_2_), 60.1 (s, 1C, *C*(CH_3_)_2_), 115.7 (s, 2C, Ar*C*), 126.1 (s, 1C, Ar*C*CH=CH), 127.6 (s, 2C, Ar*C*), 128.8 (s, 1C, ring *C*H), 129.6 (s, 1C, Ar*C*H=CH), 130.3 (s, 1C, ring CH=*C*), 135.6 (s, 1C, *C*H=CHAr), 155.2 (s, 1C, A*rC*-OH). MS (EI): *m*/*z* (%): 272 (M^+^, 42), 242 (38), 227 (72), 185 (33), 107 (100). Anal calcd. for C_17_H_22_NO_3_: C, 74.97; H, 8.14; N, 5.14. Found: 74.83; H, 8.02; N, 5.11. HO-758 was synthesized as previously described [33]. All other reagents were purchased from Merck (Darmstadt, Germany) and Molar Chemicals (Halásztelek, Hungary).

The mass spectra were recorded on a GCMS-2020 (Shimadzu, Tokyo, Japan), operated in EI mode (70 eV), and a ThermoScientific Q-Extractive HPLC/MS/MS with ESI(+) ionization (Thermo Scientific, Waltham, MA, USA). Elemental analyses were performed using a Fisons EA 1110 CHNS elemental analyzer (Fisons Instruments, Milan, Italy). The melting points were measured with a Boetius micro melting point apparatus (Franz Küstner Nachf. K. G., Dresden, Germany). ^1^H NMR spectra were collected with a Bruker Avance 3 Ascend 500 system (Bruker BioSpin Corp., Karlsruhe, Germany), operated at 500 MHz, and ^13^C NMR spectra collected at 125 MHz CDCl_3_ at 298 K. The “in situ” reduction of nitroxides was carried out by adding five equivalents of hydrazobenzene (DPPH/radical). The chemical shifts and coupling constant (J) are given in ppm and hertz, respectively. IR spectra were recorded using a Bruker Alpha FT-IR instrument (Bruker Optics, Ettlingen, Germany) with ATR support on a diamond plate. Flash column chromatography was performed using a Merck Kieselgel 60 (0.040–0.063 mm). Qualitative TLC was performed using commercially available plates (20 × 20 × 0.02 cm) coated with Merck Kieselgel (Darmstadt, Germany) GF_254_.

### 2.3. Preparation of Aqueous Aβ

A11-positive Aβ oligomers were prepared using modifications to the protocol reported by [34]. Aβ powder was dissolved at a concentration of 2.5 mg/mL in hexa-fluoro-isopropanol (HFIP). The sample was then rotated overnight and stored in aliquots (0.4 mL) at −80 °C. An aqueous solution was then made by adding the HFIP stock to a 2 mL microfuge tube, followed by the slow addition of 1 mL of 0.1 M NaHCO_3_ (pH 9.6) while stirring. HFIP was then evaporated using nitrogen gas flow over the stirred solution until the volume reached ~0.9 mL. The sample’s volume was brought to 1.0 mL with 0.1 M NaHCO_3_ (pH 9.6). The sample was then centrifuged for 10 min at 15,000 rpm to remove large amorphous aggregates, as described previously [18]. The supernatant was collected, and the removal of large aggregates was verified as the broadening of the negative 200 nm band was performed via circular dichroism [35,36]. The final concentration of peptide was ~0.9 mg/mL (estimated from absorbance at 280 nm).

Samples of oligomeric Aβ (AβO) were then made, as described previously [18], by combining the 0.9 mg/mL sample of Aβ (in 0.1 M NaHCO_3_) at a 1:1 ratio with 50 mM Tris-Borate and 150 mM NaF (pH 7.0). The sample was incubated at room temperature for 4 h while stirring with a 4 mm stir bar, and it was then diluted to the experimental concentration with 50 mM Tris-Borate (pH 7.4) and used within three hours.

### 2.4. Cell Culture Model Producing Intracellular Aβ

MC65 cells are neuronal cells derived from a human neuroblastoma line developed to accumulate intracellular Aβ [37,38,39,40]. This is achieved via the conditional expression of the carboxyl-terminal 99 residues of the amyloid-β precursor protein (APP-C99) that is suppressed by tetracycline (TC) in the culture medium (TC+). The removal of TC from cell the culture medium (TC−) induces the expression of APP-C99, which is then cleaved by the cellular γ and β secretases to generate Aβ. Intracellular Aβ is known to start to accumulate as early as 4 h after TC removal, with maximal levels reached at 24 h. Cell death relative to the TC+ control is measured 3 days after the removal of TC, with the increased death correlated with the intracellular accumulation of AβOs rather than the small amounts of secreted Aβ [40]. Cell survival was determined using an MTT assay [40]. Cells were treated with either DMSO or the indicated concentration of PAL at the same time as TC removal, with a uniform level of DMSO (0.05%) used in all assay cultures. Cell survival is expressed as the mean percentage viability of 3 × 10^4^ cells/well counted from n = 3 cultures, with parallel TC+ cultures of equal numbers of cells set at 100% viability. To determine the PAL concentration needed to achieve 50% protection, data were plotted in the Origin graphing and analysis software version 9 (OriginLab Corp, Northampton, MA, USA) using the logistic dose–response function. R^2^ values ranged from 0.982 to 0.999.

### 2.5. A11 ELISA Assay

For ELISA measurements, 60 μL of 0.2 mg/mL aqueous Aβ was added to each well of a 96-well Greiner FLUOTRAC™ 600 high-binding microplate, followed by 200 μL of freshly made 0.1 M NaHCO_3_ with pH 9.6. The plates were then incubated overnight at 4 °C. Wells were then treated with 300 μL of blocking solution (50 mM Tris buffer (pH 7.4) containing 100 g/L dried milk) for 1 h, and then washed twice with the same solution. Each well was then incubated with 300 μL of 40 μM PAL (or vehicle control) in a wash buffer (50 mM Tris buffer (pH 7.4) containing 50 g/L dried milk) for 1 h, and then washed 3× with wash buffer. Wells were then treated with 300 μL of primary antibody solution that contained the PAL or vehicle control (A11 antibody diluted 1:1200 in wash buffer containing 40 μM PAL), followed by a 2 h incubation. Wells were then washed 3× with wash buffer and incubated with HRP-conjugated secondary antibody (GAR antibody diluted 1:1200 in wash buffer). Wells were then washed 3× with wash buffer and the HRP activity was quantified busing luminescence by adding 150 μL of both SuperSignal (Thermo) chemiluminescent HRP substrate reagents to each well. We measured wells for each sample treatment in each assay in quadruplicate.

### 2.6. EPR Spectroscopy

For electron paramagnetic resonance (EPR) analysis of nitroxide redox states, PALs were exposed to conditions generating either hydroxyl or peroxyl radicals, as previously described [41], with the following modifications: PALs (160 µM final) or acetonitrile (ACN) vehicle controls were included in place of the spin trap and the reaction generating hydroxyl radicals containing 4 mM final GSH. Fifteen minutes after the reaction’s initiation, samples (~5 μL) were loaded into capillaries and scanned on a JEOL TE-100 X-band spectrometer using a loop-gap resonator [23] (JEOL USA, Peabody, MA, USA). The spectra were obtained by averaging two 2 min scans with a sweep width of 100 G at a microwave power of 4 mW and modulation amplitude that was optimized to the natural line width of the attached spin probe. All the spectra were recorded at room temperature.

### 2.7. Circular Dichroism Measurements

Circular dichroism (CD) spectroscopy measurements were performed on an aqueous AβO sample diluted with 50 mM Tris-Borate, pH 7.4, to a concentration of ~0.15 mg/mL. Samples were maintained at room temperature and CD scans were performed within 2 h of sample dilution. PALs were added from a 4 mM stock in ACN to reach a final concentration of 40 μM. CD measurements were performed on a Jasco J-715 spectropolarimeter (JASCO Inc., Tokyo, Japan) at room temperature. Samples were placed in a 1 mm pathlength quartz cuvette and CD spectra were collected by signal averaging three scans in the region 190 to 260 nm using a scan speed of 20 nm/min, bandwidth of 1 nm, and response time of 4 s. Following acquisition, all spectra were baseline-subtracted from the Tris-Borate background buffer containing either the PAL alone or the ACN solvent vehicle (the background signals were generally indistinguishable). The percentage of secondary structure was estimated by deconvolution using the BeStSel CD analysis tool [42], which can be accessed online at https://bestsel.elte.hu (accessed on 15 November 2024).

### 2.8. Thioflavin T Assay

Prior to each assay, a fresh 1 mM Thioflavin T (ThT; Sigma Aldrich, product # T3516) was prepared in cold DI water and filtered through a 0.22 µm syringe filter. ThT fluorescence was measured for 3 microliters of aqueous Aβ (~0.9 mg/mL in NaHCO_3_) added to the wells of a black, Nunc MicroWell 384-well nonbinding optical bottom microplate (cat # P9241-30EA), which was then added to 50 μL of PBS buffer. Samples were then treated with PALs (18 μM) or DMSO vehicle control. Assays were initiated with 1 μL of ThT (20 µM final) and fluorescence was measured at room temperature, using a TECAN Infinite 200Pro plate reader, Tecan, Mennedorf, Switzerland, through the bottom of the plate, with excitation at 440 nm and emission reading at 486 nm. Intensity data were collected for 24 h at 5 min intervals. Background correction samples contained no Aβ and were subtracted from samples containing Aβ. For the untreated Aβ control sample, the fluorescence intensity of ThT in PBS was used for background subtraction. For Aβ+PAL samples, the background of ThT+PAL was subtracted from samples containing Aβ.

### 2.9. Nile Red Assay

Prior to each assay, a fresh solution of 1 mM Nile Red (Sigma Aldrich, product # 19123) was prepared in DMSO and centrifuged at 10,000 RPM to remove any aggregates. Nile Red fluorescence was measured using 3 microliters of aqueous Aβ (~0.9 mg/mL in NaHCO_3_) added to the wells of a black, Nunc MicroWell 384-well nonbinding optical bottom microplate (cat # P9241-30EA), which was then added to 50 μL of PBS buffer. Samples were treated with PALs (18 μM final) or vehicle control. Assays were initiated with 1 μL of Nile Red. Nile Red fluorescence was measured at room temperature, using a TECAN Infinite 200Pro plate reader, through the bottom of the plate, with emissions readings of 558 nm and 635 nm. Intensity data were collected for 24 h at 5 min intervals. Background correction samples contained no Aβ and were subtracted from samples containing Aβ. For the untreated Aβ control sample, the fluorescence intensity of Nile Red in PBS was used for background subtraction. For Aβ+PAL samples, the background of Nile Red+PAL was subtracted from samples containing Aβ.

### 2.10. Measurements of Relative ROS Scavenging

The hydroxyl radical scavenging activity of our PALs was determined by measuring the relative fluorescence of 2′,7′-dichlorofluorescein (DCFH) with PAL compared to the control of DCFH in the master mix. Hydroxyl radicals were generated via a Fenton reaction from a mix of 65% DCFH (80 µM), 9% MES buffer (100 mM), 9% glutathione (GSH) (4 mM), 5% CuCl_2_ (10 mM), and 7% acetonitrile (ACN), acting as a control, or 7% ACN+PAL to give a final PAL concentration of 40 µM. The master mix was vortexed and, to the remaining 5% of the mix, 0.3% H_2_O_2_ was added and the mix was vortexed again. The reaction mix was incubated at room temperature for 15 min and then diluted 1:20 in 100 mM MES buffer. The control sample and PAL samples were measured in a fluorescence spectrometer, where λ_ex_ = 485 nm and λ_em_ = 510–530 nm. The peak was determined to be 521.94 nm and data points corresponding to that wavelength were used for analysis.

The superoxide radical scavenging of our PALs was assessed by measuring the fluorescence of DCFH with PAL relative to the control of DCFH in the master mix. A GSH+ master mix of PBS pH 7.4, 0.11% HRP (10 mg/mL), 0.11% DCFH (20 mM), and 5.6% GSH (80 mM) was created to measure the fluorescence of DCFH. Another master mix was created using the same concentrations listed above, albeit with no GSH added, to create a baseline to subtract from the GSH+ mix. Master mixes were added to a 96-well plate in triplicate, using 3 GSH+ and 3GSH−, for each PAL or control. PALs and controls were added to their respective wells and then mixed for 10 s in a plate shaker. Then, 0.3% H_2_O_2_ was added to every well and mixed by pipette 3× before taking the reading. The final contents of each well were 45 µL of master mix and 1 µL of ACN or 1 mM of PAL and 5 µL of H_2_O_2_. Fluorescence was read from the bottom, with λ_ex_ = 485 nm and λ_em_ = 535 nm, gain = optimal, and settle time was 10 msec, as in the settings. Triplicates were averaged and GSH- average was subtracted from the GSH+ average and then compared to the GSH+ corrected control.

### 2.11. Animals and Animal Treatment

We purchased 5×FAD transgenic female mice (MMRRC Stock 34848; B6.Cg-Tg (APPSwFlLon,PSEN1*M146L*L286V)6799Vas/Mmjax) from Jackson Laboratory (Bar Harbor, ME, USA) and housed them at the UC Davis Teaching and Research Animal Care Services (TRACS) facility under the direction of the campus Attending Veterinarian in compliance with all ethical and humane care guidelines.

For mouse dosing, an aqueous treatment formulation of PMT-307 was used, consisting of 2.9% PEG-400, 2.9% Tween-80, and 4.5% ethanol, with a PAL concentration of 2.5 mM. The treatment of mice commenced at 11 weeks of age. Mice were treated twice weekly at a dose of 4 mg/kg (s.c.) for a period of 24 weeks.

### 2.12. Tissue Harvesting and Preparation

Following the PMT-307 treatment period, animals were transferred to the UC Davis Comparative Pathology Laboratory (CPL) for tissue harvesting and sectioning. Mice were euthanized and brains were removed and dissected at the midline. Right hemibrains were reserved for homogenization and biochemical analyses via immediate snap freezing in liquid nitrogen and storage at −80 °C until use. Left hemibrains were drop-fixed in 4% paraformaldehyde (PFA) for 24 h at 4 °C. They were then dehydrated and placed in 30% sucrose in 0.01 M PBS until the hemibrain sank to the bottom of the container. The hemibrains were frozen on dry ice and coronal sections were cut to 10 μm thickness on a sliding microtome. The free-floating sections were stored at −20 °C in cryoprotectant (30% ethyleneglycol, 25% glycerol, 25% 0.1 M PBS, and 20% distilled water).

### 2.13. Multiplex Immunohistochemistry

Formalin-fixed, paraffin-embedded brain tissue sections, cut to a thickness of 4 microns, were mounted on APEX BOND adhesive slides (Leica Biosystems, Nußloch, Germany) and stained using the Leica BondRX autostainer (Leica Biosystems) by modifying the process reported previously (PMID: 33590360). The primary antibodies used were anti-NeuN (1:1000; Novus Biologicals, Centennial, CO, USA, NBP1-77686) and anti-MOAB-2 (1:1000; Novus Biologicals, NBP2-13075). Detection and signal amplification were achieved with the Opal anti-MS+RB HRP secondary antibody (Akoya Biosciences, Marlborough, MA, USA) and tyramide signal amplification. We used Opal-520 and Opal-690 to visualize each marker. DAPI was used for nuclear counterstaining. During staining optimization, we assessed pre-existing tissue autofluorescence, primary antibody, and Opal dye concentrations, by following previously reported protocols (PMID: 33590360). The optimization process was evaluated by examining signal-to-noise ratios and signal balance in the tissue images, which were scanned using the Vectra/Polaris multispectral imaging system (Akoya Biosciences) and analyzed with inForm software version 2.6.0 (Akoya Biosciences).

### 2.14. Immunohistochemistry (IHC)

The brain sections were bathed in 0.01M PBS, containing 0.3% Triton-X100 (PBST). The specimens were incubated with a 10 mM sodium citrate buffer for 30 min at 85 °C for antigen retrieval and then cooled for 30 min at room temperature. After antigen retrieval, the brain sections were washed again with PBST and incubated at room temperature for 20 min in 0.1 M sodium metaperiodate to quench endogenous peroxidase activity. Sections were then washed twice in PBST containing 10% horse serum to block non-specific reactions. Alternate brain slices were then treated overnight at 4 °C (using the vendor’s recommended dilution) with primary antibody anti-IbA-1 (Novus Biologicals; NB100-1028) or primary antibody anti-4-HNE (R&D Systems, Minneapolis, MN, USA; MAB3249) in a solution of PBST containing 1% horse serum. Subsequently, sections were washed in PBST + 1% horse serum (3 × 10 min), which was incubated (1 h, room temp) with biotinylated secondary antibody (anti-mouse IgG for 4-HNE or anti-goat for IbA-1) at a dilution 1:200. The samples were then washed (PBS 3 × 10 min) and then incubated with the avidin–biotin complex (1:500; Elite Kit, Vector Labs, Newark, CA, USA) for 1 h. Sections were bathed in a 0.2 M sodium acetate trihydrate and 1.0 M imidazole solution (pH 7.4 with acetic acid; 3 × 10 min). Signals were visualized using an acetate–imidazole buffer containing 0.05% 3/3′-diaminobenzidine tetrahydrochloride (DAB) and 0.0015% hydrogen peroxide for 15 min. Sections were bathed in acetate–imidazole buffer (3 × 5 min), transferred to PBS, mounted onto glass slides, air-dried overnight, dehydrated through increments of alcohol content (70%, 95%, 100%; 3 × 5 min), cleared in xylene (3 × 5 min), and cover-slipped with DPX (BDH Laboratory Supplies, Poole, UK).

### 2.15. Image Analysis

Scanned im3 files from the Vectra/Polaris multispectral imaging system were converted into multilayered TIFF files using inForm software and subsequently analyzed in QuPath version 0.4.4 (PMID: 29203879). The TIFF files were changed into an OME-TIFF format to reconstitute the entire tissue image within QuPath. Regions of interest, including the cortex, hippocampus, and thalamus, were annotated, with at least three areas marked for each lesion type in each tissue sample. QuPath’s cell detection function was employed for cell segmentation, capturing data on each marker’s intensity within both the cytoplasm and nuclei of individual cells. GraphPad Prism version 7.0e (San Diego, CA, USA) was then used for data visualization and statistical analysis.

### 2.16. Cytokine Measurements

Cytokine levels were determined by their mRNA expression using quantitative RT-PCR (qRT-PCR) at the UC Davis Real-time PCR Research and Diagnostics Core Facility (Davis, CA, USA). Measurements were performed on a small specimen of cortical brain tissue collected from the flash-frozen right hemisphere, with the remaining tissue preserved for biochemical analyses (see Section 2.17 and Section 2.18). Tissue samples were collected and frozen at −80 °C. A small piece of tissue (20 mg) was added to a solution of 400 µL of ATL and 40 µL Proteinase K containing two grinding beads (4 mm diameter, stainless steel beads, SpexCertiprep, Metuchen, NJ, USA). Tissues were homogenized in a GenoGrinder2000 (SpexCertiprep) for 2 min at 1000 strokes per minute. Total nucleic acid was extracted from the tissue lysates using the DNeasy Blood and Tissue Kits from Qiagen (Qiagen, Valencia, CA, USA) according to the manufacturer’s instructions.

For each target gene, two primers and an internal hydrolysis fluorescent labeled probe (5’ end, reporter dye FAM (6-carboxyflourescein), 3′ end, quencher NFQMGB (Non-Fluorescent Quencher Minor Grove Binding)) were purchased from Thermo Fisher (Thermo Fisher Scientific, Carlsbad, CA, USA). The Quantitect Reverse transcription kit (Qiagen) was used for cDNA synthesis following the manufacturer’s directions with the following modifications. Overall, 10 microliters of RNA were digested with 1 μL of gDNA WipeOut Buffer (5 min, 42 °C) and briefly centrifuged. Genomic DNA contamination was tested by using 1 μL of digested RNA and the real-time PCR housekeeping gene. Then, 0.5 μL of Quantitect Reverse Transcriptase, 2 μL Quantitect RT buffer, 0.5 μL RT Primer Mix, and 0.5 μL 20 pmol Random Primers (Invitrogen, Waltham, MA, USA) were added. The reaction volume was adjusted to 20 μL and the solution was incubated at 42 °C for 40 min. The samples were inactivated at 95 °C for 3 min, chilled, and diluted with the addition of 80 μL DIH_2_O. The quantity of applied RNA was simultaneously normalized to cDNA samples that had been amplified for glyceraldehyde-3-phosphate dehydrogenase (GAPDH)-specific primers.

Primers for specific mouse cytokine genes were obtained from ThermoFisher, catalog # 4331182. (IL-1b, Assay ID Mm00439614_m1; TNF-a, Assay ID Mm00443258_m1; IL-10, Assay ID Mm00439614_m1). Each PCR reaction used the commercially available PCR mastermix (TaqMan Universal PCR Mastermix, Applied Biosystems, Waltham, MA, USA), using the 20× primer and probes for the respective qPCR systems, with a final primer concentration of 400 nM, an 80 nM concentration of the TaqMan probe, and 5 μL of the diluted cDNA sample for a final volume of 12 μL. The samples were placed in 384-well plates and amplified in an automated fluorometer (QuantStudio 7 Pro, ABI). Standard ABI amplification conditions were used: 2 min at 50 °C, 10 min at 95 °C, 40 cycles of 15 s at 95 °C, and 60 s at 60 °C. Fluorescent signals were collected during the annealing temperature period and Cq values were extracted with a threshold of 0.1 and baseline values of 3–10. The final quantitation was calculated using the comparative Cq method (User Bulletin #2, Applied Biosystems) and this was reported as the n-fold difference relative to the calibrator cDNA (i.e., lowest target gene transcription). In brief, the three reference genes were averaged to normalize the Cq values of the target genes (ΔCq). The ΔCq was calibrated against the average of the negative control group within each target gene. The linear amount of target molecules relative to the calibrator was calculated by 2^−ΔΔCq^. Therefore, all gene transcription is expressed as an n-fold difference relative to the calibrator.

Statistical analysis: Calculated values for gene expression changes were obtained using GraphPad PRISM software version 7.0e (San Diego, CA, USA). An unpaired Student’s *t* test was used to compare treatment groups for significant differences. Differences with *p* ≤ 0.05 were considered significant. Results are expressed as MEAN ± SEM.

### 2.17. Tissue Homogenate Preparation and Western Blot Analysis

The right brain hemispheres were divided in half, with one half used for Western blot analysis and the other half used for ELISA analysis (Section 2.18). Remaining tissues were homogenized in a lysis buffer (150 mM NaCl, 10 mM NaH_2_PO_4_, 1 mM EDTA, 1% Triton X-100, 0.5% SDS) with a protease inhibitor cocktail and a phosphatase inhibitor (Sigma, St. Louis, MO, USA). Equivalent amounts of protein were analyzed via 4–20% Tris-Glycine gel electrophoresis (Invitrogen). Proteins were transferred to PVDF membranes and probed with antibodies at 4 °C overnight. Visualization was enabled using enhanced chemiluminescence (Amersham, Buckinghamshire, UK). The following primary antibodies (dilutions) were used: anti-phosphoERK (1:1000, Cell Signaling, Danvers, MA, USA, #4370), anti-phospho-CaMKII (1:1000, Cell Signaling, #12716), and β-actin (1:5000, Cell Signaling, #3700). For secondary antibodies, we used HRP-conjugated anti-rabbit (1:1000, Cell Signaling, #7074) or anti-mouse antibodies (1:1000 and 1:5000, Cell Signaling, #7076).

### 2.18. Tissue Homogenate Preparation and Aβ ELISA

Brain homogenates samples were fractionated into Tris-buffered saline (TBS)-soluble and TBS-insoluble and SDS-soluble fractions. Briefly, brain tissues were homogenized in TBS with protease inhibitors, followed by centrifugation (100,000× *g*, 1 h, at 4 °C). The supernatants were collected as the TBS-soluble fraction. The pellets were homogenized in 2% SDS with protease inhibitors. This was followed by centrifugation (100,000× *g*, 1 h, at 4 °C). The supernatants were collected as the TBS-insoluble, SDS-soluble fraction. Each fraction was then measured for Aβ42 levels using the Aβ42 ELISA kit (Invitrogen).

## 3. Results

**Figure 2:**
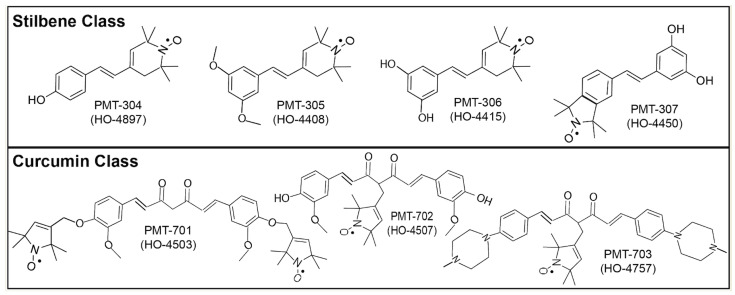
The chemical structure of PAL candidates. For convenience, PAL family codes are designated as PMT. The original HO- designations, as previously published, are also provided.

The structures of the PALs are shown in Figure 2. The method behind the synthesis of PMT-304 is provided in the Section 2. The synthesis of the other stilbene PALs (PMT-305-307) was previously reported [32]. The synthesis of the curcumin analogs PMT-701-703 was previously reported [31]. Using the SwissADME tool [43], the physicochemical properties of each PAL were calculated (Table 1). These properties can inform us about the ability of small molecules to function as CNS therapeutics [43]. On the basis of this analysis, each of the stilbene PALs is predicted to penetrate the blood–brain barrier (BBB) (Table 1). In contrast, the larger polar surface area of the curcumins 701 and 702 reduces their likelihood of BBB penetration.

### 3.1. PALs Protect Neurons Against Aβ Toxicity

**Figure 3:**
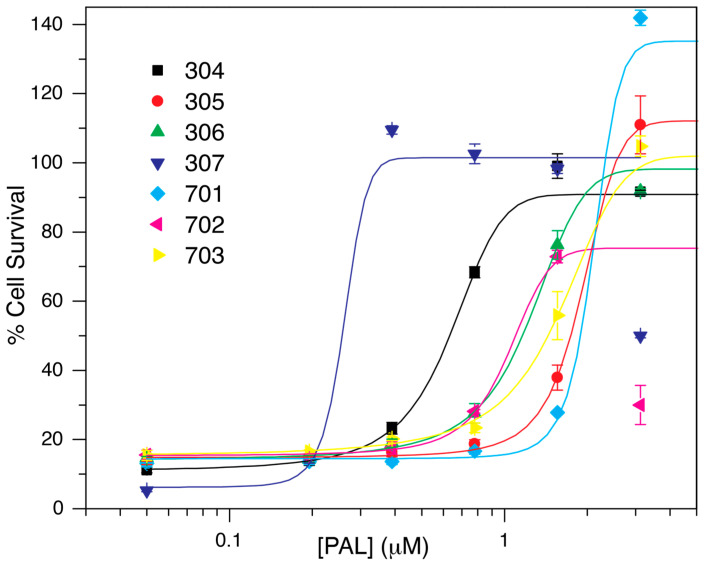
PAL neuronal protection activity. Induced (TC–) C99 expression in the MC65 model results in amyloid beta cytotoxicity that can be eliminated with PAL titration. Error bars are the SEM from the assay of three separate measurements.

The MC65 neuronal cell model was employed to identify the protection against Aβ toxicity shown by our compounds [44]. MC65 is a genetically engineered human neuroblastoma cell line that conditionally expresses a partial (amino-17 residues + carboxyl-99 residues) fusion of the APP protein with the removal of tetracycline (-TC), where C99 is expressed and cleaved by secretases to generate the Aβ peptide. This is followed by neuronal death after 3 days [45]. Previous MC65 screenings of anti-oligomerization agents showed the neuronal protection correlates closely with the level of intracellular AβOs and oxidative stress [19,20,21,46]. The protection of MC65 cells against Aβ toxicity is shown in Figure 3. The data were fitted to a dose–response function, with the calculated PC50 values given in Table 1. The stilbene candidates are more potent in the MC65 assay, with PMT-304 and -307 providing the highest potencies.

### 3.2. PAL Modulation of Aβ Structure

**Figure 4:**
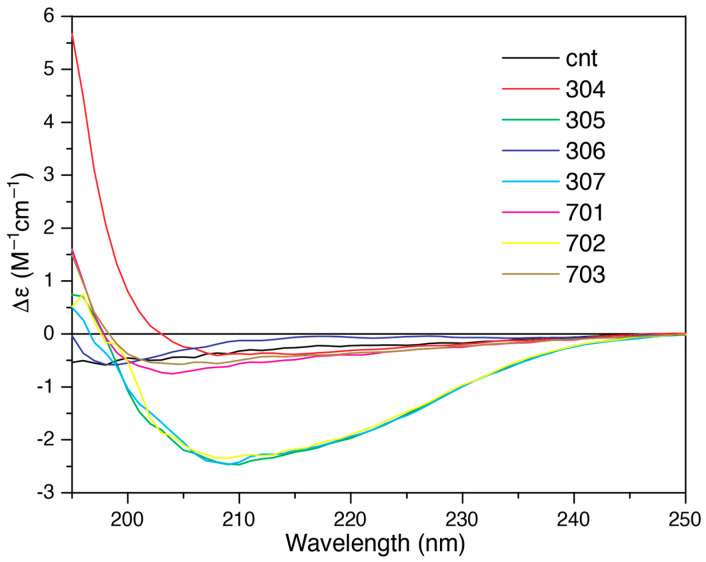
CD spectra of AβOs treated with a stoichiometric amount of PAL agent. CD spectra of AβOs treated for 1 h with 40 μM PAL. The control (black) was treated with an equal volume of vehicle. ∆ε represents the molar circular dichroism.

The activity of PALs in MC65 neuronal protection assay may reflect their ability to alter the conformation of the Aβ target, but their potency will be governed by their intrinsic properties, affecting cell penetration, intracellular distribution, and metabolic stability. To directly evaluate the ability of PALs to modulate the AβO structure, circular dichroism (CD) was used to measure the PAL effects on the secondary structure of AβOs. The characteristic hypochromic CD spectrum of the untreated AβO sample is shown in Figure 4 (black trace). These non-fibrillar early oligomers are recognized by the A11 antibody and contain low beta-sheet contents [18,23,47]. Their hypochromic spectra have been postulated to arise from a high degree of alpha-sheet content [48], although the lower CD amplitude may also reflect protein aggregation in the absence of a predominant secondary structure [49]. PAL addition strongly increases the overall CD amplitude, with the exception of PMT-306. However, the CD spectrum of PMT-306 is unique in that it appears to contain influences from a broad positive band in the region of 205–230 nm. Within this region, both turns [50] and conformations, exhibiting an equal molar ratio of PPII and a β_I_ structure, generate similar spectra [51].

The AβO sample treated with PMT-304 generates the most unusual spectrum, where its high positive intensity in the 195–200 nm range (typical for α-helical and β_I_/β-antiparallel regions [42,52]) is not accompanied by a strong negative band in the 215–220 nm regime. A unique conformation that strongly cancels CD intensity in the 215–220 range may account for the resulting spectrum in the presence of PMT-304. This notion is consistent with the strong attenuation of A11 recognition by PMT-304 (described below). Unique electronic transitions arising from the Aβ/PMT-304 complex can also be considered as a possible source of the unusual AβO CD spectrum following PMT-304 treatment. As described in [52], achiral extrinsic chromophores can generate optical activity if their interaction with a protein selects a favored conformation for the ligand. PMT-304 may also generate optical activity by combining with transitions on protein chromophores via coupled-oscillator interactions or by mixing in the electrostatic field of the protein [52].

Table 2 shows that the estimation of the fractional secondary structure was performed using the BestSel algorithm [42]. The fraction of antiparallel beta strand is a common conformational arrangement that has been identified in neurotoxic Aβ oligomers [53,54,55,56,57]. Compared to this parameter, the CD results have some correlation with PALs’ potency in cell protection. For example, the PALs with the highest and lowest PC50 values (307 and 701, respectively) are predicted to have the lowest and highest fraction of antiparallel β-sheet content. However, this effect is not observed universally, as PMT-304 displays potency in the cell protection assay without causing a decrease in the calculated antiparallel content of AβOs. As always, populations of secondary structures, calculated by deconvolution, must be viewed as estimates, and quantifying a twisted antiparallel β structure against a background of high disorder carries a large degree of uncertainty [58]. Thus, deconvolution may not capture the antiparallel β structure observed in CD spectral change accompanying PMT-304 treatment. In this regard, Misconai et al. [58] propose distinguishing samples on the basis of order/disorder using a model-independent assessment of spectral amplitudes at 197, 206, and 233 nm. The results of the order/disorder classification are included in Table 1. Notably, only the untreated control and the sample treated with PAL-306 remain largely disordered according to this metric.

### 3.3. PAL Effect of Fluorescent Dye Binding

**Figure 5:**
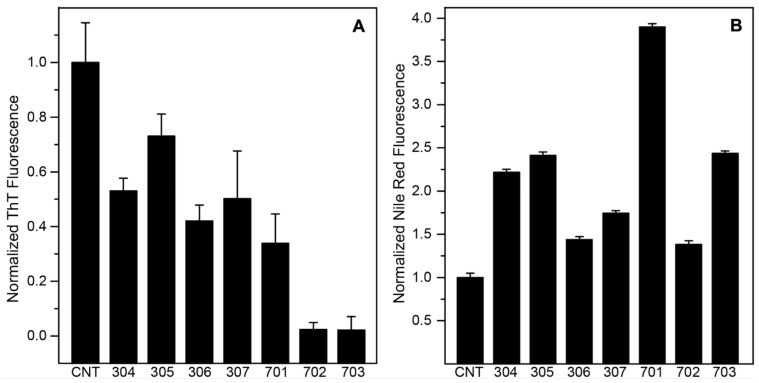
PALs’ effect on AβO-associated dyes. (**A**) The ThT fluorescence intensity of Aβ+PAL samples normalized to the sample of Aβ alone. (**B**) The NR fluorescence intensity of Aβ+PAL samples normalized to the sample of Aβ alone. Values represent the average intensities of triplicate samples measured at 20 h.

We also screened for compounds that alter oligomer structure and/or assembly according to fluorescent dye intensity, which is sensitive to beta-sheet accumulation and accessible hydrophobic surface area [59]. The fluorescent ThT dye is sensitive for the progressive increase in β-sheet content occuring with Aβ fibril assembly [60,61]. The increase in β-sheet content is therefore indicative of Aβ fibril assembly and its conformational convertion. We measured the effects of PALs on ThT fluorescence in the presence of Aβ over 24 h of incubation. As shown in Figure 5A, all of the PALs reduced the ThT intensity relative to control. This suggests that PAL binding to Aβ inhibits β-sheet formation. However, the CD results demonstrate that PALs 305, 307 and 702 induce β-sheet structures in early Aβ oligomers; thus, competition for ThT binding within β-pleated regions of AβOs must be considered for these compounds (vs. allosteric effects that reduce β-sheet formation).

To further evalaute PAL structural influences on AβOs, we screened for their effect on Nile Red (NR) fluorescence. NR fluorescence increases upon binding to available hydrophobic surfaces and can therefore provide additional evidence for PAL-induced conformational changes [62]. Relative to untreated AβOs, all PALs increase NR fluorescence, suggesting the greater accessibility of hydrophobic patches within the peptide (Figure 5B). This finding is consistent with the disruption of hydrophopbic interactions involving the central cores (LVFFA; residues 17–21 in Aβ) of neighboring peptides. This is a reasonable assumption, as the LVFFA core comprises the predicted binding location for small molecules containing aromatic rings [44,63,64,65].

### 3.4. PALs Reduce Binding of the Conformation-Specific Antibody A11

**Figure 6:**
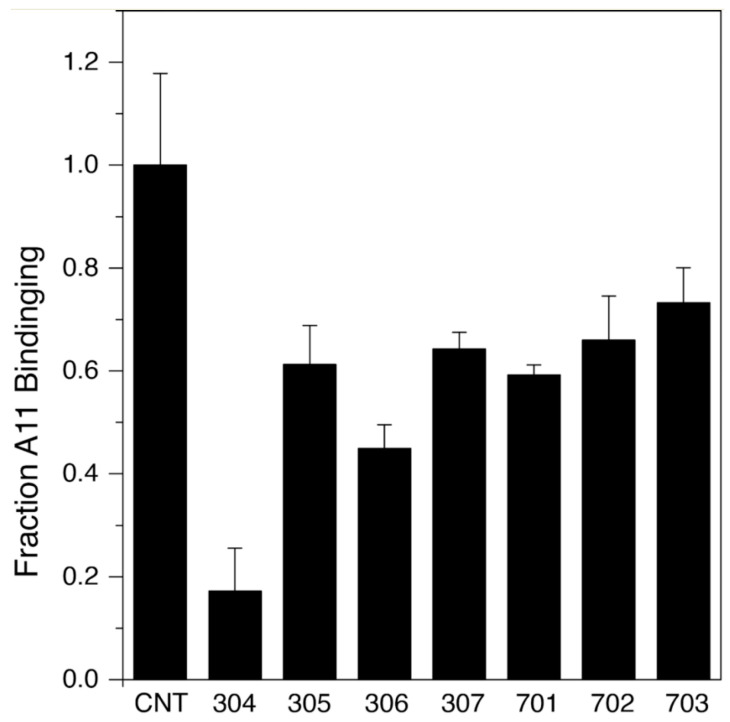
Decreases in the A11 antibody recognition of AβOs following PAL treatment. Amounts of captured A11 are reported from the oxidized luminol signal of the HRP-secondary antibody. Samples were tested in quadruplicate in each assay. The results are the average of three independent assays with errors reported as SEM.

The conformation-specific A11 antibody, identified by Glabe and co-workers [6], recognizes oligomers of disparate proteins, involved in neurodegeneration, and provides a tool for moving the modulation of the AβOs away from their “toxic” conformation [66]. We have previously used A11 to confirm the PAL-induced conformational adaptation of oligomeric amyloid beta [23]. To evaluate the effect of the PAL agents examined here on A11 recognition, we measured A11 binding to immobilized AβOs with and without PAL treatment. As shown in Figure 6, treating immobilized AβOs with a stoichiometric amount of the PAL agent significantly reduces A11 capture by 30–70%. PMT-304, the second most potent part of the MC65 protection assay, is very effective at reducing A11 binding. PMT-304 appears to be the most effective at reducing the amount of A11 recognition in this assay.

### 3.5. PAL Antioxidant Capacity

**Figure 7:**
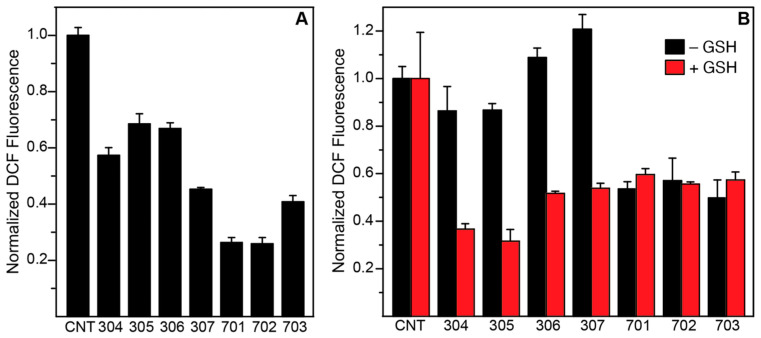
Relative antioxidant capacities of PAL candidates. The oxidation of DCFH generates a strong fluorescence reflective of ROS levels. (**A**) shows the DCF intensities following peroxidase-dependent DCFH oxidation (by O_2_•−), in the presence of PALs, relative to the ACN control. The effect of PALs on DCFH oxidation (relative to the ACN control) in the presence of •OH radical generation is shown in (**B**). A PAL concentration of 20 μM was used to compare the superoxide oxidation of 20 μM DCFH. A PAL concentration of 40 μM was used to compare the hydroxyl radical oxidation of 50 μM DCFH. All assays were performed in quadruplicate.

PALs composed of the nitroxide–stilbene and nitroxide–curcumin hybrids each have two distinct chemical species (nitroxyl and its aromatic scaffold), with each portion displaying antioxidant activity. The catalytic properties of nitroxides result from their ability to react with HO_2_• to form the oxoammonium state, which can subsequently be reduced back to the nitroxide state by reacting with the anionic O_2_•− radical [67]. Because neither of the three redox states of the nitroxide are damaging to biomolecules, nitroxides can provide SOD mimetic activity with a net conversion of O_2_•− into H_2_O_2_+O_2_ [67] (Figure 1). To compare the relative antioxidant capacity of each candidate, we used the ROS-sensitive dye 2′,7′-dichlorodihydrofluorescein (DCFH). DCFH is a non-fluorescent probe that produces intense fluorescence upon oxidation to 2′,7′-dichlorofluorescein (DCF) [68]. The suitability of the PALs examined here to serve as antioxidants was verified by their ability to reduce the oxidation of the DCFH indicator in the presence of peroxidase or hydroxyl radicals. In the peroxidase system, the oxidation of DCFH can occur along two pathways. First, there is the direct oxidation of DCFH by peroxidase enzyme, forming the DCF• radical, which then reacts with oxygen to form fluorescent DCF [68]. Second, DCFH oxidation may occur through reactions with superoxide (O_2_•−) decomposition products (e.g., via the Haber–Weiss reaction [69]). Antioxidants can therefore be screened for their ability to repair the DCF• radical and/or decrease levels of superoxide decomposition products that can directly oxidize DCFH, such as the hydroxyl radical and singlet oxygen [68,70]. As shown in Figure 7A, at 16 μM, each of the PALs attenuated the HRP/H_2_O_2_ conversion of DCFH into DCF, with the curcumin-based PALs being more effective as a group. As nitroxides [71], phenolic stilbenes [72], and curcumin [73] can directly interact with superoxide, their ability to reduce levels of O_2_•− and thereby its decomposition products likely contributes to the observed antioxidant activity in the peroxidase system.

The ability of the PALs to reduce the oxidation of DCFH with hydroxyl radical (•OH) generation is shown in Figure 7B. Nitroxides react poorly with •OH, but can oxidize them to an oxoammonium state [74]. However, •OH reacts with both curcumin and stilbenes, and so most direct scavenging is likely elicited from the scaffold portion of the PAL. As a group, the stilbene PALs do not reduce DCFH oxidation by •OH when reduced glutathione (GSH) is omitted. The GSH dependence of the stilbene polyphenols has been previously described: in the absence of GSH, polyphenolic stilbenes enhance the Cu(II)-catalyzed production of •OH [75]. This phenomenon is consistent with the fact that PMT-306 and -307 increase DCFH oxidation relative to the control in the absence of GSH. However, in the presence of reducing agents such as ascorbate or GSH, phenolic stilbenes switch to displaying antioxidant activity in the presence of •OH radicals generated by the Fenton reaction [75]. The rapid conversion of •OH into thiyl radical GS• can be effectively scavenged by both stilbenes [75] and nitroxides [76]. Indeed, in the presence of GSH, the antioxidant activity of each stilbene PAL is observed (Figure 7B). Thus, in the cellular environment of millimolar GSH, each of the stilbene PALs is expected to reduce the oxidative damage elicited by •OH. In contrast, each of the curcumin PALs, at 8 μM, strongly reduces DCFH oxidation, independently of GSH. This is consistent with curcumin’s established capacity to scavenge •OH directly [77].

**Figure 8:**
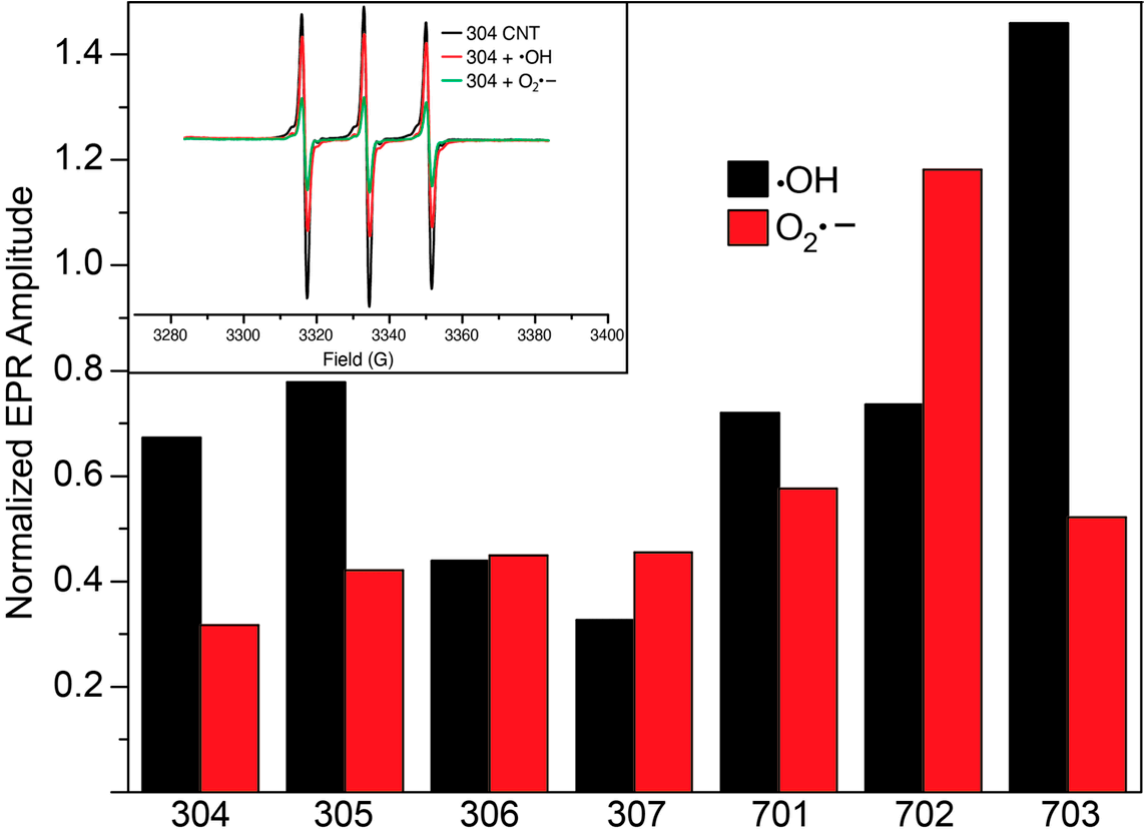
Effect of ROS on PAL nitroxide signal as measured by EPR spectroscopy. Shown are center line amplitudes of PAL nitroxide (160 μM) following incubation with either O_2_•− or •OH radical relative to PAL in buffer. Reduction in EPR amplitude is indicative of oxidation of N-oxyl moiety to diamagnetic state. We included 4 mM GSH in •OH radical samples to diminish differential production of Cu(II)-catalyzed •OH radical generation in stilbene PALs.

The involvement of the nitroxyl moiety in the peroxidase and Fenton ROS measurements was probed by EPR for both reaction conditions (Figure 8). In these measurements, the loss of paramagnetism demonstrates the participation of the nitroxyl moiety in the redox chemistry, with conversion into either the reduced hydroxylamine or the oxidized oxoammonium resulting in a diamagnetic species with no EPR signal (see above). With the exception PMT-702, all the candidates show nitroxide redox activity in the presence of O_2_•− production (Figure 8). In the case of PMT-702, the net increase in the signal with O_2_•− suggests that the reductive capacity of the curcumin moiety maintains an equilibrium of nitroxide and its hydroxylamine, which is then shifted towards nitroxyl upon reaction with O_2_•−.

As discussed above, the direct reaction of the nitroxide with the •OH radical (to form the oxoammonium state) is comparably less efficient than that with O_2_•− (Figure 8). Notably, PMT-703 undergoes a large increase in paramagnetic state upon •OH radical production, reflecting a shift in the N-OH/N-O equilibrium towards the N-O species. The intensity above the control for PMT-703 likely reflects the reaction of its residual hydroxylamine population, with transient radicals captured in the scaffold portion of the PAL (see the radical oxidation of hydroxylamine in Figure 1). In this regard, the piperazine rings unique to PMT-703 have been shown to efficiently form radicals in the presence of •OH [78]. Thus, piperazine ring radicals provide a way to fully oxidize residual hydroxylamine and increase the net EPR signal intensity. In summary, the results of these measurements demonstrate that the nitroxyl moieties of each PAL participate in the candidate’s antioxidant activity, although the direct reaction with •OH is limited relative to that with O_2_•−.

### 3.6. Evidence of In Vivo Activity

To test for evidence of PALs’ therapeutic activity, we carried out a pilot in vivo study on the candidate showing the highest potency in the MC65 assay (PMT-307). For this purpose, we utilized the 5xFAD mouse model, an aggressive Alzheimer’s model that carries 5 mutations responsible for familial AD pathology and rapidly develops amyloid pathology, with deposition beginning as early as 1.5 months and plaque formation found across the hippocampus and cortex by 6 months of age [79]. Here, we compared three treated 5xFAD female mice to three 5xFAD control mice injected with vehicle alone. Given the small study size, only female mice were used, as this sex displays a more profound phenotype [80,81]. As mouse tolerance to PMT-307 had not been determined, we selected a relatively low dose of 4 mg/kg with twice-weekly subcutaneous (s.c.) administration over 24 weeks, starting at 10 weeks of age, with control animals injected with vehicle. At the end of the study period, brains were harvested, and the left hemisphere was used to obtain sections for Aβ, NeuN, Iba1, and 4-HNE staining (using immunofluorescence or IHC (Iba1 and 4-HNE). The other hemisphere was homogenized for biochemical analyses to assess the levels of soluble Aβ and synaptic signaling pathway proteins.

Immunofluorescence was used to determine levels of Aβ and the neuronal-specific marker NeuN. No significant difference in total amyloid (in both cortex and hippocampus) was observed. However, mice treated with PMT-307 showed lower levels of intracellular Aβ relative to controls, although the difference was only significant for NeuN(-) cells in the cortex (Figure S1A). The hippocampal (HC) region showed a reduction vs. controls for the PMT307-treated animals. In addition, NeuN staining of the cortical region (CX) found that drug treatment significantly increased the ratio of neuronal cells relative to glial cells, suggesting that the compound reduces neuronal loss and/or neuroinflammation (Figure S1B).

Immunohistochemistry (IHC) was used to detect levels of Iba1 and 4-HNE staining. The levels of Iba1, an inflammatory marker for activated microglia, were measured in both the treated and control animals. PMT-307 treatment resulted in a reduction of Iba1 in the hippocampal region, but this effect did not reach statistical significance in readings performed on cortical tissue (Figure S1C). To look for evidence of PAL treatment lowering oxidative stress, brain sections were also stained for protein adducts of the cytotoxic aldehyde 4-Hydroxynonenal (4-HNE), a lipid peroxidation product released during the oxidation of ω-6-unsaturated fatty acids. Consistent with a previous study on 4-month-old 5xFAD mice [82], relatively low levels of 4-HNE staining were found in both cortical and hippocampal regions. Unexpectedly, mice treated with PMT-307 displayed a small, but significant increase in 4-HNE staining (Figure S1D).

The right brain hemisphere was further probed by biochemical analysis. First, qPCR was carried out to examine the levels of cytokines in the two groups. The major inflammatory cytokines (IL-1b and TNF-α) did not show a statistical difference between the two groups. Interestingly, animals treated with PMT-307 had a 3.9-fold increase (*p* < 0.01) in IL-10 (Figure S2A), an anti-inflammatory cytokine that down-regulates pro-inflammatory responses in microglia [83]. As previous studies have associated enhanced IL-10 expression with neuroprotection in AD animal models [83,84], PMT-307 may offer benefit by inhibiting inflammatory cytokine release in the brain.

The brain homogenates were also measured to assess levels of Aβ42 [85] via ELISA. Mice treated with PMT-307 showed a slight overall reduction in SDS-soluble Aβ; however, the difference versus the control group did not reach the level of statistical significance (Figure S2B). Finally, we also probed the effect of PMT-307 on the activation of both ERK (extracellular signal-regulated kinase) and CaMKII (Ca^2+^/Calmodulin-dependent protein kinase II). These enzymes play a pivotal role in synaptic plasticity, learning, and memory in the hippocampus, and their downregulation correlates with cognitive impairment and AD progression [86,87]. We therefore probed the phosphorylation status of both proteins in the control and treated groups [85]. While the treated group showed higher overall phosphoprotein levels, the differences did not reach the level of significance (Figure S2C–G).

## 4. Discussion

The AβO species represents a challenging drug target, as their structures are ill defined. Multiple lines of evidence correlate toxicity with oligomers, encompassing a dynamic equilibrium of sizes that contain a high degree of conformational disorder [10]. This intrinsic disorder probably underlies the ability of Aβ to disrupt multiple cellular processes; therefore, molecules that convert the peptide into states that are either non-toxic or more readily cleared may counteract Aβ’s conformational toxicity [88].

In vivo, a plethora of factors relating to AβO toxicity have been identified, including familial mutations, the ratio of Aβ40/Aβ42, and posttranslational modification [89]. In vitro studies have probed for specific conformational markers such as turns, secondary structures, intra- and intermolecular contacts, hydrophobic surface exposure, and the relative orientation (parallel or antiparallel) of the peptides in the assembly [53,89]. For example, AβO recognition by the A11 antibody has been correlated with the presence of antiparallel β-sheets [53,89]. However, the transient nature of AβOs, arising from both a high degree of disorder within the peptide as well as the dynamic exchange of constituents between oligomers, has prevented any definitive structural state from governing the interaction of AβOs with cellular targets. Nevertheless, the ability of the A11 antibody to recognize pathogenic oligomers formed by unrelated proteins suggests that a common conformational feature underlies the ability of these assemblies to disrupt cellular homeostasis.

Intrinsically disordered targets are challenging to deal wih from a drug design perspective. Our previous studies show that PALs stabilize Aβ oligomers to have a narrower size distribution, while maintaining a high level of backbone disorder [18,20]. This effect may seem counterintuitive with respect to the thermodynamic prediction that PALs reduce oligomer toxicity by enhancing the secondary structure and increasing overall order. On this point, Vendruscolo and co-workers have proposed a mechanism for the small-molecule attenuation of oligomer toxicity by the stabilization of the “native” intrinsically disordered conformation of Aβ [90]. By retaining the conformational entropy of the peptide backbone through transient non-specific interactions, a small molecule–AβO ensemble can achieve a lower energy state than a more organized species (e.g., pre-fibrillar oligomers with a high β-sheet content).

The compounds studied here fall within a diverse collection of planar aromatic compounds that inhibit Aβ oligomer and/or fibril formation [63,65,66,91,92], sharing a common feature in that they possess small molecules adept at interrupting protein–protein interactions [93]. The distinct polar and apolar regions found in each of the compounds are also properties associated with effective anti-amyloid small molecules [94]. Generalizing their structural commonalties (phenolic groups separated a rotationally constrained linker) suggests these small molecules induce a conformational adaptation of Aβ involving interactions at spatially distinct sites within the hydrophobic core of the peptide [64,65,92]. Furthermore, the fact soluble Aβ remains largely disordered after PAL engagement is consistent with a dynamic interaction along the peptide surface [95]. Our goal is to use PALs to chaperone the outcome of this process, serving as structural correctors that shift the dynamic Aβ ensemble to a less pathogenic state [96], while also localizing antioxidant functionality to prevent the oxidative potentiation of AβO toxicity [24,25,26].

An important caveat relating to the correlation of biophysical measurements of PAL effects on AβOs with their potency in the cell model must be considered, as the later will also be influenced by the PALs’ cell penetration, intracellular distribution, and metabolic stability. Of the PALs evaluated here, PMT-307 acts with the highest potency in the neuronal cell assay. The fused ring structure of PMT-307 is unique among the PALs evaluated here, a feature that offers greater interaction with the peptide’s central hydrophobic region [64,92]. It is also the only stilbene with a five-membered nitroxide, meaning it is likely to undergo a slower rate of bioreduction to hydroxylamine compared to the piperidine versions [97,98]. PMT-305, the only stilbene that lacks a hydroxyl group, is the least potent stilbene PAL. In countering Aβ toxicity, the stilbene phenolic groups are postulated to play a role in both the compound’s antioxidant activity and Aβ binding [64,99,100]. Furthermore, PMT-305 is the most hydrophobic stilbene, having the highest iLOGP value (Table 1). Thus, the diminished distribution (e.g., out of the cell membrane) may also play a role in its lower potency. As for the curcumin PALs, PMT-702 displays the highest potency. This finding is consistent with previous studies, as PMT-702 maintains the functional groups of native curcumin. Specifically, the substitution of the free hydroxyl groups on the aromatic rings greatly reduces the ability of curcumin to inhibit Aβ fibril assembly [63].

Our pilot in vivo study suggested the therapeutic activity of PMT-307 in the 5xFAD model; however, the observed effects were mild, and only a few parameters had statistical significance. A larger sample size, along with the use of pharmacokinetic data to inform on dosing levels, will enable a more rigorous determination of the therapeutic potential of these molecules. As found in previous studies in animals and humans, which have not reported acute toxicity to be associated with nitroxides [101], no adverse clinical findings were observed in the treated animals, which is encouraging from a safety perspective.

The significant difference in intraneuronal Aβ levels between the treated and control 5xFAD mice is encouraging, as the ability of small molecules to engage pathogenic intraneuronal AβOs [11] may be advantageous compared to immunotherapeutic approaches. Unexpectedly, the reduction of intracellular Aβ was most significant in the NeuN(-) cell population. However, modulating non-neuronal Aβ may represent a consequential AD intervention. A recent report shows that oligodendrocytes contribute to the primary AD pathology via Aβ production, including a significant role in the overall plaque burden [102].

Although staining of the oxidative stress marker 4-HNE was low in both groups, the finding of increased 4-HNE levels in the treated animals is paradoxical given the antioxidant capacity of PMT-307 measured in vitro. As the IHC detects only the His-conjugated HNE adduct, we cannot be certain that this finding reflects differences in total 4-HNE production, which is regulated by multiple reactions within the cell [103]. Nevertheless, this finding points to the need for further studies on PMT-307 metabolism and to probe for other markers of oxidative stress.

## 5. Conclusions

In summary, PAL compounds can have a potential disease-modifying effect by interfering with two recognized features associated with the progression of AD arising from the pathogenic conformation of intra- and extracellular AβOs, and by attenuating oxidative stress. Developing small molecules with bifunctionality provides meaningful advantages for the development and administration of small-molecule therapeutics. Because in vivo safety and efficacy testing (e.g., tolerance, pharmacokinetics, pharmacodynamics) are carried out for single agents, there is growing interest in developing compounds to address more than one target [104,105,106], including interest in targeting both amyloid assembly and oxidative stress [107]. More extensive measurements are needed on the ability of PALs to synergistically modulate two pathogenetic hallmarks of AD. This dual-target approach may potentially enhance the overall pharmacological effect, increasing the prospects of disease modification for neurodegenerative indications.

## Figures and Tables

**Table 1:** Physicochemical properties of PALS and PC50 of PAL candidates. The total polar surface area (TPSA) and the implicit log of the octanol/water partition coefficient (iLOGP) were calculated using SwissADME [43]. These values, along with other physicochemical properties, were employed with the SwissADME tool (http://www.swissadme.ch; accessed on 15 October 2024) to predict blood–brain barrier penetration (BBB_pen_). The measured cell protection potency (PC50) was determined by fitting the data to a logistic dose–response function.

PAL	MW (g/mol)	TPSA (Å^2^)	iLOGp (o/w)	BBB_pen_ Prediction	PC_50_ (mM)
PMT-304	272.4	43.70	3.17	YES	0.63
PMT-305	316.4	41.93	3.97	YES	1.92
PMT-306	288.4	63.93	2.69	YES	0.94
PMT-307	324.4	63.93	2.85	YES	0.26
PMT-701	672.8	123.66	6.05	NO	2.10
PMT-702	519.6	119.36	4.59	NO	1.04
PMT-703	624.8	73.40	5.29	YES	1.24

**Table 2:** Results from the fitting of the CD spectra by the BeStSel algorithm to estimate fractions of secondary structure in Aβ samples.

Structure Class	cnt	304	305	306	307	701	702	703
α	0.0	1.3	13.0	2.1	13.0	0.0	8.1	0.0
β	37.2	41.0	21.8	36.1	23.3	41.4	32.4	41.0
β_antipara_	37.2	41.0	17.3	36.1	1.7	41.4	25.8	41.0
turn	16.7	15.1	13.5	14.9	15.3	18.0	13.2	17.6
other	46.1	42.5	51.7	47.0	48.3	40.5	46.4	41.5
ordered (O) disordered (D)	D	O	O	D	O	O	O	O

## Data Availability

The data that support the findings will be available in Dryad at datadryad.org/stash following an embargo from the date of publication to allow for commercialization of research findings.

## Supplementary Materials

The following supporting information can be downloaded at: https://www.mdpi.com/article/10.3390/antiox13111411/s1, Figure S1: Histopathological analysis of 5xFAD brain sections; Figure S2: Biochemical analysis of 5xFAD brain hemispheres.
