# Supplementary material for: Nitroxyl Hybrids with Curcumin and Stilbene Scaffolds Display Potent Antioxidant Activity, Remodel the Amyloid Beta Oligomer, and Reverse Amyloid Beta-Induced Cytotoxicity
Source: Antioxidants (Basel). 2024 Nov 18;13(11):1411. doi: 10.3390/antiox13111411 (PMC11591036; doi:10.3390/antiox13111411)

Supplementary Material

Figure S1. **Histopathological analysis of 5xFAD brain sections**. Shown are the results compiled from six female mice: three controls and three treated with PMT-307. Panel **A** shows levels of intracellular Aβ in the cortex of control (C) and treated (T) 5x-FAD mice. Levels are shown for cells with and without association to the neuronal-specific marker NeuN. For ***; $p<0.001$. Intensities were measured by immunofluorescence. Panel **B** shows the effect of drug treatment on the NeuN+/NeuN- ratio of cells in the cortex of study animals. For *; $p<0.05$. Intensities were measured by immunofluorescence. Panel **C** shows the level of the inflammatory marker Iba1 in cortex (CX) and hippocampus (HC) of control (C) and treated (T) 5x-FAD mice. Staining was measured by IHC. For **; $p<0.01$. Panel **D** shows the levels of oxidative stress marker 4-HNE in cortex (CX) and hippocampus (HC) of control (C) and treated (T) 5x-FAD mice. Staining was measured by IHC. For *; $p<0.05$ and for **; $p<0.01$.

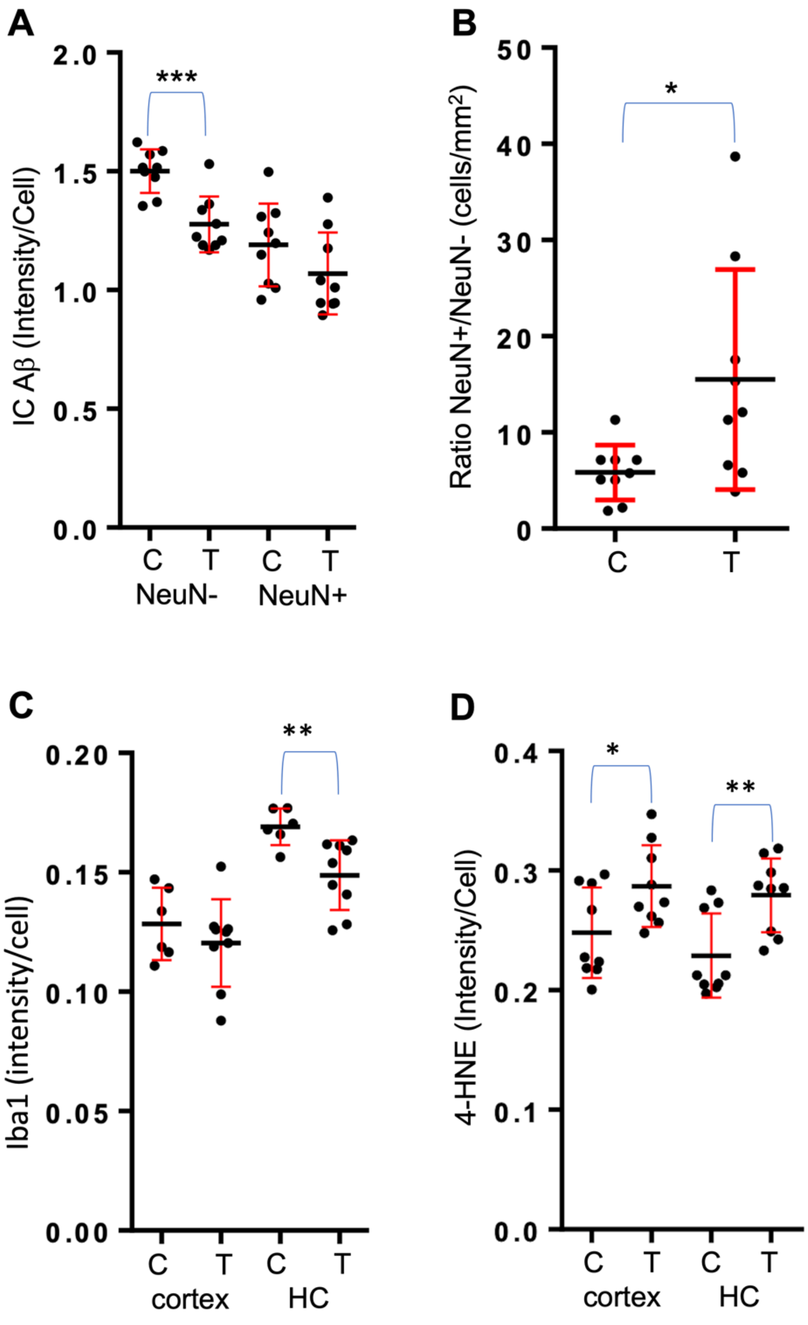

Figure S2. **Biochemical analysis of 5xFAD brain hemispheres**. Shown are the results compiled from six female mice: three controls and three treated. (**A**) qPCR quantification of pro-inflammatory (IL-1B and TNF-α) and anti-inflammatory cytokine (IL-10) RNA expression in brain. Plotted is the fold-change (qPCR) of cytokine levels in the treated compared the control mice. For **; p<0.01. (**B**) The brains were fractionated into SDS-soluble fractions, which were used for ELISA quantification of A*β*42. n=3, unpaired t-test. (**C, D, E, F, G**) Representative images of Western blots for phospho-ERK p42/p44 and phosphor-CaMKII p50/p60 and quantification of band intensity expressed as fold change. *n* = 3, unpaired t-test.

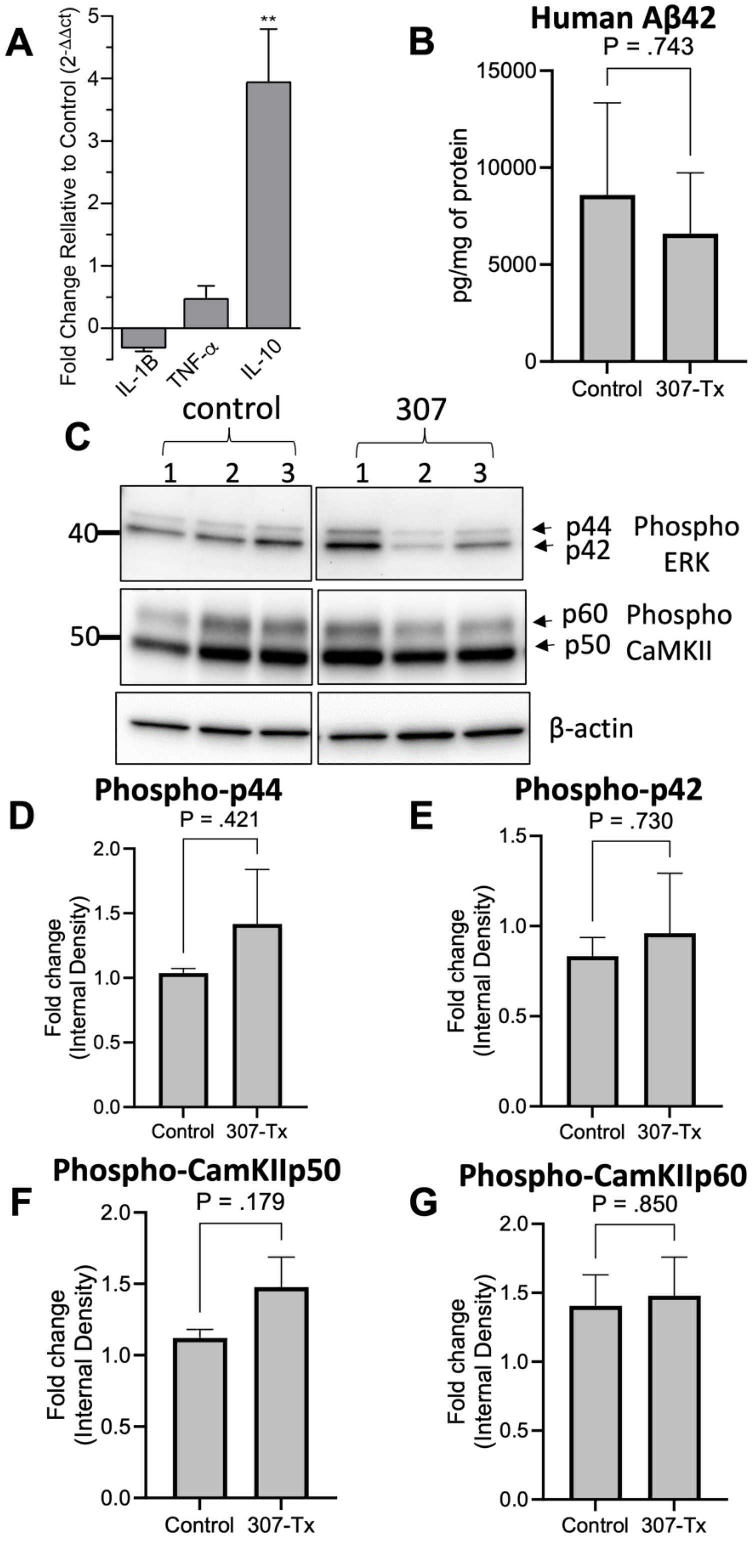

Supplement: Supplementary file 1 [file antioxidants-13-01411-s001.zip › antioxidants-3280561-supplementary.pdf]
